# Linked-evidence modelling of qualitative G6PD testing to inform low- and intermediate-dose primaquine treatment for radical cure of *Plasmodium vivax*

**DOI:** 10.1371/journal.pntd.0012486

**Published:** 2024-09-05

**Authors:** Michelle L. Gatton

**Affiliations:** Centre for Immunology and Infection Control, Faculty of Health, Queensland University of Technology, Brisbane, Australia; Swiss Tropical and Public Health Institute: Schweizerisches Tropen- und Public Health-Institut, SWITZERLAND

## Abstract

**Background:**

Radical cure of *Plasmodium vivax* infections is key to the control of vivax malaria. However, the standard doses of 8-aminoquinoline drugs used for radical cure can cause severe haemolysis in G6PD-deficient patients. The availability of near-patient G6PD tests could increase use of primaquine (PQ), however direct evidence of the impacts that G6PD testing has on downstream patient outcomes, such as haemolysis and recurrence is lacking.

**Methodology/Principle findings:**

A linked-evidence model was created to investigate changes in the number of severe haemolysis events and *P*. *vivax* recurrences within 6 months of treatment when qualitative G6PD testing was used to guide PQ treatment (0.25mg/kg/day for 14 days and 0.5mg/kg/day for 7 days), compared to prescribing 14-day PQ with no G6PD testing. In the model patients identified as G6PD-deficient received 8-week PQ (0.75mg/kg/week). The model was used to simulate scenarios with 1%, 5% and 10% prevalence of G6PD-deficiency (G6PDd) in theoretical populations of 10,000 male and female *P*. *vivax* patients and initially assumed 100% adherence to the prescribed PQ regiment. Results illustrate that G6PD testing to guide the 14-day PQ regiment reduced severe haemolysis by 21–80% and increased recurrences by 3–6%, compared to applying the 14-day PQ regiment without G6PD testing. Results for the 7-day PQ regiment informed by G6PD testing were mixed, dependent on G6PDd prevalence and sex. When adherence to the PQ regiments was less than perfect the model predicted reductions in the number of recurrences at all prevalence levels, provided adherence to 7-day PQ was 5–10% higher than adherence to the 14-day regiment.

**Conclusions/Significance:**

Introduction of G6PD testing to guide PQ treatment reduces severe haemolysis events for the 14-day regiment, and the 7-day regiment in higher G6PDd prevalence settings, compared to use of 14-day PQ without G6PD testing when all patients adhere to the prescribed PQ treatment. At a population level, there were increases in recurrences, but this could be resolved when the 7-day regiment was used and had superior adherence compared to the 14-day regiment.

## Author summary

To eliminate vivax malaria treatment is required that cures the blood infection and also kills any dormant parasites in the liver (called hypnozoites). Unfortunately, drugs currently available to treat hypnozoites such as primaquine can cause severe haemolysis when given to patients who have glucose-6-phosphate dehydrogenase (G6PD) deficiency, a common human enzyme polymorphism. The author created a mathematical model that compares the number of severe haemolysis events and vivax recurrences within 6 months of treatment in a theoretical population when qualitative G6PD tests are used to guide primaquine treatment versus the use of primaquine without consideration of the G6PD status of the patient. The results demonstrate that adding G6PD tests into the clinical pathway can reduce the number of severe haemolysis events, but may increase the number of recurrences, with specific results determined by the prevalence of G6PD deficiency, the dosing regiment and adherence to the prescribed primaquine treatment.

## Introduction

*Plasmodium vivax* remains the cause of significant morbidity in many countries in South East Asia, Western Pacific, Americas and the Eastern Mediterranean [1]. Control of *P*. *vivax* presents a public health challenge due to the ability of the parasite to cause relapses after initial infection. It is estimated that at least 79% of *P*. *vivax* infections are attributable to relapses [2], highlighting the importance of reducing the dormant hypnozoite reservoir responsible for relapses.

The 8-aminoquinoline drugs primaquine (PQ) and tafenoquine are used for the radical cure of *P*. *vivax*. However, all 8-aminoquinoline drugs cause oxidant haemolysis in people with glucose-6-phosphate dehydrogenase (G6PD) deficiency [3]. The World Health Organization (WHO) guidelines for radical cure of *P*. *vivax* recommend the use of PQ, with the exact regiment dictated by the G6PD status of the patient and local policy [4]. Reviews of national policies on the use of PQ for radical cure and G6PD testing published in 2018 and 2023 show substantial variation across malaria endemic countries, and highlight a gap between policy and practice [5,6].

G6PD deficiency (G6PDd) is a common human enzyme polymorphism associated with the *g6pd* gene located on the X chromosome. Males are either G6PD-deficient or G6PD-normal, while females can be deficient, intermediate or normal in G6PD activity [7]. Different mutations in the *g6pd* gene, or variants, produce a range of G6PD activity phenotypes. The population allele frequency of G6PDd varies between countries from <0.5% to >20%, with an estimated median frequency in malaria endemic countries of 8.0% [5,8]. G6PD activity is a continuum and is commonly expressed as a percentage of ‘normal’ activity. For consistency between studies, ‘normal’ activity has been defined as the median G6PD activity in males, excluding G6PD-deficient males, and is referred to as the adjusted-male median (AMM) [9]. Individuals with G6PD activity <30% of the AMM are typically classified as having a severely deficient phenotype [7]. Although population estimates exist for G6PDd allele frequency, the prevalence of the severely deficient phenotype among malaria patients is unknown but likely lower than in the general population due to the protective effect of G6PDd against malaria infection [10].

The increasing availability of near-patient qualitative and semi-quantitative G6PD tests provides additional opportunities for G6PD testing to guide PQ treatment. These tests are cheaper, faster and more accessible than ultraviolet spectrophotometry, the reference diagnostic method for determining G6PD activity. Cost-effectiveness analysis for settings in Brazil, Lao DPR and Thailand have indicated that near-patient quantitative [11,12] and qualitative [13,14] G6PD testing strategies are cost effective for clinical complications requiring hospitalisation in G6PD-deficient patients [11,13] and disability-adjusted life years averted [12,14]. These studies help address one of the perceived barriers to implementing G6PD testing in routine care [15].

There have been numerous trials assessing the performance of near-patient G6PD tests, but the true impact of these tests on patient outcomes is felt downstream after treatment has been administered. While it seems logical to assume that implementing G6PD testing is beneficial, there is no direct evidence of the impact using currently available near-patient G6PD tests has on patient outcomes such as haemolysis and relapse. To address this gap the current study developed a linked-evidence model to address the research question “In individuals with *P*. *vivax*, what is the impact on patient morbidity of using qualitative G6PD tests to guide PQ treatment decisions, compared to using PQ with no G6PD testing?”

## Methods

### Analytical framework and sources of evidence

**Fig 1:**
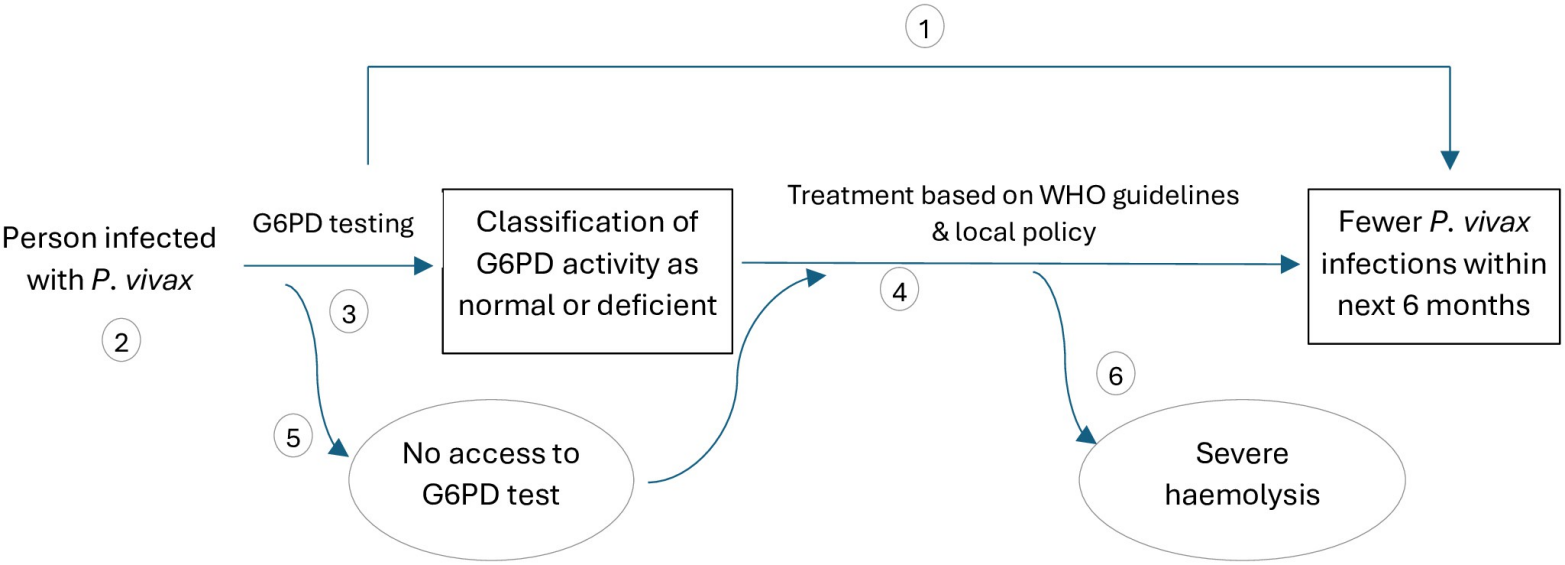
Analytical framework for using a qualitative G6PD test to guide PQ treatment decisions. [1] Is there direct evidence that using a qualitative G6PD test reduces *P*. *vivax* infections in the 6 months following treatment? [2] What is the prevalence of G6PD activity <30% of adjusted-male median in the target group? [3] Can G6PD tests be used to accurately classify G6PD activity? [4] Does treatment with PQ result in fewer future *P*. *vivax* infections? [5] What are the consequences of not having access to near patient G6PD testing? [6] Does treatment result in adverse effects, specifically severe haemolysis?

A linked-evidence modelling approach was employed based on the analytical framework in Fig 1. Evidence to inform the model was primarily sourced from systematic reviews and/or meta-analyses (Table 1).

**Table 1:** Sources of evidence and parameter values used to inform the linked-evidence model.

Question	Evidence and Source	*Parameter name in model* & distribution [low, high]^a^
1. Is there direct evidence that using a qualitative G6PD test reduces *P*. *vivax* infections in the 6 months following treatment?	No direct evidence exists	
2. What is the prevalence of G6PD activity <30% of AMM in the target group?	Estimated median G6PDd allele frequency in malaria endemic countries is 8.0%, with marked variability in country-specific estimates from <0.5% to >20% [8].	
(a) What proportion of males have G6PD activity <30% of AMM?	1.0% at 1% G6PDd allele frequency 5.0% at 5% G6PDd allele frequency 10.0% at 10% G6PD allele frequency [7]	*Dist* [14] *~* Dirichlet Dirichlet(97.0;2.0;1.0) Dirichlet(93.0;2.0;5.0) Dirichlet(88.1;1.9;10.0)
(b) What proportion of females have G6PD activity <30% of AMM?	0.2% at 1% G6PDd allele frequency 1.1% at 5% G6PDd allele frequency 2.5% at 10% G6PDd allele frequency [7]	*Dist* [14] *~* Dirichlet Dirichlet(93.0;6.8;0.2) Dirichlet(87.6;11.3;1.1) Dirichlet(80.6;16.9;2.5)
3. Can G6PD tests be used to accurately classify G6PD activity?	The majority of studies assessing the performance of near-patient G6PD tests use ultraviolet spectrophotometry as the reference diagnostic method. Evidence shows that the G6PD activities reported by spectrophotometry are site-specific and subject to inter-replicate, and intra- and inter-laboratory variability [16]. Variations in repeatability could result in misclassification of individuals, the magnitude of which is dictated by the proportion of individuals with G6PD activity near the classification threshold. However, in the absence of a perfect test to assess G6PD activity, it is assumed that the rate of misclassification using spectrophotometry is negligible.	
(a) What is the sensitivity of qualitative G6PD tests at <30% G6PD activity?	Estimate from systematic review and meta-analysis of the lateral flow assay from Access Bio/CareStart (8 studies): 0.96 (95% CI 0.90–0.99) [17].	*G6PD_sn ~* Beta(112.0,7.0) [0.75–1.00]
(b) What is the specificity of qualitative G6PD tests at <30% G6PD activity?	Estimate from systematic review and meta-analysis of the lateral flow assay from Access Bio/CareStart (8 studies): 0.95 (95% CI 0.92–0.96) [17].	*G6PD_sp ~* Beta(279.8, 10.9) [0.90–1.00]
4. Does treatment with PQ result in fewer future *P*. *vivax* infections?	Meta-analysis (23 studies from 16 countries) revealed mean incidence rate of *P*. *vivax* recurrences between days 7 and 180 post-treatment was 1.84 recurrences per person-year (95% CI 1.74–1.95) in patients not receiving PQ [18].	*Recur_no_pq ~* Norm(1.844, 0.055)/2 [0.75–1.00]
(a) Does treatment with 0.25 mg/kg PQ daily for 14 days reduce *P*. *vivax* infections in the next 6 months? (Total PQ dose of 3.5 mg/kg)	Meta-analysis (23 studies from 16 countries) revealed mean incidence rate of *P*. *vivax* recurrences between day 7 and 180 post-treatment was 0.47 recurrences per person-year (95% CI 0.42–0.52) in patients receiving a total dose of 2—<5 mg/kg PQ [18].	*Recur_pq ~* Norm(0.470, 0.025)/2 [0.20–0.275]
(b) Does treatment with 0.50 mg/kg PQ daily for 7 days reduce *P*. *vivax* infections in the next 6 months? (Total PQ dose of 3.5 mg/kg)	See 4(a) above. Systematic review found no difference in *P*. *vivax* recurrences at six to seven months post-treatment for 0.5 mg/kg/day for seven days compared to 0.25 mg/kg/day for 14 days [19].	
(c) Does treatment with 0.75 mg/kg PQ weekly for 8 weeks reduce *P*. *vivax* infections in the next 6 months?	Multi-centre clinical trial (four countries) showed reduced risk of recurrence 12-months following treatment: incidence rate 0.047 per person-year (95% CI 0.012–0.187, n = 50) vs 1.32 per person-year (95% CI 1.15–1.48, n = 464) for placebo [20]. However, patient distribution between study sites differed for patients receiving 8-wk PQ and placebo, with study sites having different relapse periodicity and annual parasite index. Results are not comparable to recurrence rate for 7- or 14-day PQ, or no PQ, reported in row 4 and 4(a) above due to different follow-up period and no statistical adjustment for potential confounders.	*Recur_8wk_pq ~* Norm(0.785, 0.032)/2 [0.20–1.00]
5. What are the consequences of not having access to near patient G6PD testing?	Local policy is applied. In the model this is 14-day PQ without G6PD testing.	
6. Does treatment result in adverse effects, specifically severe haemolysis^b^?		
(a) What is the rate of severe haemolysis following treatment with 0.25 mg/kg PQ daily for 14 days in individuals with normal G6PD activity?	For patients with G6PD activity > 30%, meta-analysis (18 studies from 15 countries) revealed incidence of severe haemolysis was 0.0% (0/893; 95% CI 0.0–0.4%) [21]	*Hem_risk_n_low ~* Beta(1,893) [0.00–0.004]
(b) What is the rate of severe haemolysis following treatment with 0.5 mg/kg PQ daily for 7 days in individuals with normal G6PD activity?	For patients with G6PD activity > 30%, meta-analysis (18 studies from 15 countries) revealed incidence of severe haemolysis was 0.3% (5/1464; 95% CI 0.1–0.8%) [21]	*Hem_risk_n_mod ~* Beta(5,1459) [0.00–0.008]
(c) What is the rate of severe haemolysis following treatment with 0.75 mg/kg PQ weekly for 8 weeks in individuals with normal G6PD activity?	Limited data. No severe haemolysis detected in 9 Indonesian patients with (measured) G6PD activity >30% AMM [20].	
(d) What is the rate of severe haemolysis following treatment with 0.25 mg/kg PQ daily for 14 days in individuals with deficient G6PD activity?	A review of 20 studies that included confirmed G6PD-deficient individuals indicated 11.2% (27/241) of G6PD-deficient individuals experienced severe adverse events following daily PQ, but PQ dosage was not specified [3]. Most severe adverse events were haemolysis, with or without a requirement for blood transfusion and results were across different PQ dosing regiments.	*Hem_risk_d_low ~* Beta(27,241) [0.05–0.25]
(e) What is the rate of severe haemolysis following treatment with 0.5 mg/kg PQ daily for 7 days in individuals with deficient G6PD activity?	See 6(d) above	*Hem_risk_d_mod ~* Beta(20,80) [0.10–0.50]
(f) What is the rate of severe haemolysis following treatment with 0.75 mg/kg PQ weekly for 8 weeks in individuals with deficient G6PD activity?	Overall incidence of severe adverse events following single or weekly PQ doses (irrespective of G6PD status) was 0.42% (16/3,771) (95% CI 0.22–0.63) [3]. Likely that all adverse events occurred in G6PD-deficient individuals, however the number of G6PD-deficient patients within the studies is unknown so no incidence rate can be calculated. No severe adverse events following weekly PQ amongst 41 patients (4 countries) who were G6PD deficient according to fluorescent spot test and had confirmed G6PD activity <30% AMM or did not have G6PD activity measured [20].	*Hem_risk_d_8wk ~* Beta(1,99) [0.00–0.04]

^a^ Distributions have means and 2.5^th^ and 97.5^th^ percentiles that match mean and 95% confidence intervals reported in literature; low and high values included only for variables included in one-way sensitivity analysis

^b^ Severe haemolysis is defined as a haemoglobin reduction of more than 25% to a concentration <7 g/dL between day 0 and days 1–14 post-treatment

AMM: Adjusted male-median

### Outcomes of model

The outcomes used to reflect patient morbidity within a population of 10,000 treated *P*. *vivax* patients are the number of recurrences within 6 months of treatment and the number of PQ-induced severe haemolysis events. The number of recurrences can be considered an indicator of the effectiveness of the testing and treatment protocol (where fewer recurrences are better), while the number of severe haemolysis events is indicative of the human health cost of the treatment protocol. The 6-month follow-up period for recurrences was selected as this period has been shown to have the highest hazard of hospital admission and death upon reinfection with *P*. *vivax* [22].

Literature indicates that *P*. *vivax*-infected individuals can experience severe haemolysis without PQ treatment [21]. For this reason, the model specifically focuses on additional severe haemolysis events that arise when individuals are treated with PQ.

There is little consensus in the literature regarding the definition of severe haemolysis. In this study severe haemolysis is defined as a reduction in haemoglobin of more than 25% to a concentration of less than 7 g/dL between day 0 and days 1–14 post-treatment. This definition targets severe adverse events likely to require medical intervention (eg blood transfusion).

### Population included

Only individuals who are potentially eligible for treatment with PQ are considered in the model. Excluded individuals include pregnant women, infants aged < 6 months and women breastfeeding infants aged < 6 months. These individuals are excluded since the G6PD testing strategy adopted will not impact the treatment they receive. Separate simulations of the model were conducted for males and females due to differences in the prevalence of G6PDd.

### Treatments applied within the model

The model incorporates PQ treatment as per the WHO guidelines [4]:

1. 14-day course of PQ at 0.25 mg/kg body weight per day for individuals known to have normal G6PD activity (low-dose PQ);
2. 7-day course of PQ at 0.5 mg/kg/body weight per day for individuals known to have normal G6PD activity (intermediate-dose PQ);
3. 8-week course of PQ at 0.75 mg/kg body weight once per week for individuals with known G6PDd (8-wk PQ).

The definitions of G6PD-deficient and G6PD-normal in the context of PQ treatment guidelines refer to <30% AMM G6PD activity and ≥30% AMM G6PD activity, respectively.

Within the model, when the G6PD status of a patient is unknown the low-dose PQ regiment is administered.

### Assumptions

Assumptions need to be made in the model when relevant evidence does not exist. These include:

1. Individuals who have a severe haemolysis event stop PQ treatment and therefore do not benefit from the reduced rate of *P*. *vivax* recurrence that results after complete PQ treatment. These individuals are assumed to have the same recurrence rate as individuals who do not receive PQ.
2. The rate of severe haemolysis in G6PD-deficient patients is highest with intermediate-dose PQ (20%), followed by low-dose PQ (10%), followed by 8-wk PQ (1%).
3. There is no increased risk of severe haemolysis for G6PD-normal individuals on the 8-wk PQ regiment.
4. The mean number of *P*. *vivax* recurrences in the 6 months following the 8-wk PQ regiment is 0.43-fold the mean number of *P*. *vivax* recurrences when PQ is not administered. This assumption reflects literature that supervised 8-wk PQ treatment is effective, compared to no PQ [20].

### Model structure and parameters

The model predicts the occurrence of the two outcomes for two different comparative scenarios.

1. Implementing qualitative G6PD testing to guide low-dose PQ treatment, in comparison to low-dose PQ treatment without G6PD testing. In this comparison the only difference between scenarios is the use of qualitative G6PD testing and 8-wk PQ where G6PDd is indicated by the G6PD test. Compared to patients receiving low-dose PQ, patients taking 8-wk PQ have an assumed lower risk of severe haemolysis and a higher expected number of recurrences within 6 months of treatment.
2. Implementing qualitative G6PD testing to guide intermediate-dose PQ treatment, in comparison to low-dose PQ treatment without G6PD testing. In this comparison G6PD testing is coupled with use of intermediate-dose PQ in patients who return a normal G6PD test or 8-wk PQ in patients identified as G6PD-deficient by the G6PD test, versus the baseline scenario where all patients receive low-dose PQ. The evidence outlined in Table 1, combined with the model assumptions, result in there being a higher risk of severe haemolysis in both G6PD-normal and G6PD-deficient patients when intermediate-dose PQ is used rather than low-dose PQ, which has a higher risk of severe haemolysis than 8-wk PQ. The expected number of recurrences within 6 months of treatment is assumed to be the same for intermediate-dose and low-dose PQ, but lower than for 8-wk PQ.

**Fig 2:**
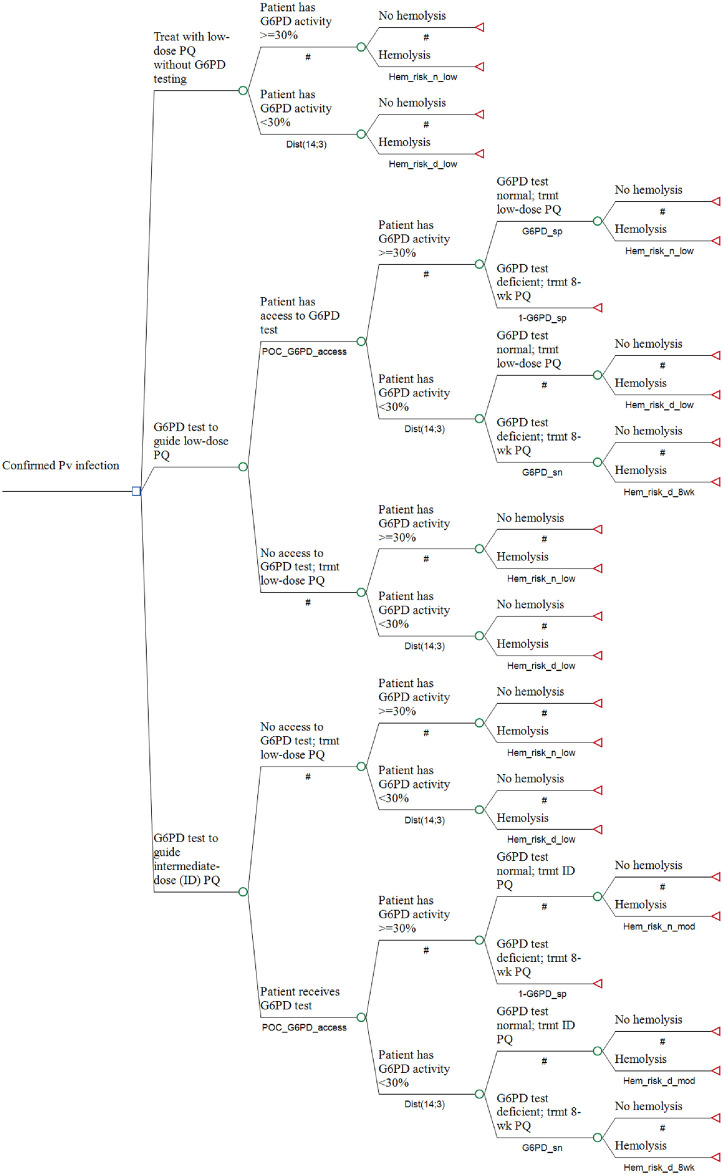
Model structure. Probabilities are defined in Table 1. ID: intermediate-dose.

In both comparative scenarios administration of low-dose PQ without G6PD testing is the baseline. The model structure is presented in Fig 2, while the parameter distributions for each parameter are presented in Table 1. The parameter distributions were guided by available evidence, with the mean and central 95% of the distributions reflecting the mean and 95% confidence intervals reported in the literature.

One of the key modifiable factors in the analysis is the proportion of the population who have access to G6PD testing. Different simulations were conducted for access levels ranging from 0% to 100% of the population having access to the G6PD test; 0% access represents the baseline scenario where no patients have access to a G6PD test.

### Implementation and sensitivity analysis

The model was created and implemented using TreeAge Pro 2023 (Healthcare Version) (TreeAge Software, LLC) [23]. Separate versions of the model were created for males and females at 1%, 5% and 10% prevalence of G6PDd. A one-way sensitivity analysis of the male model at 1% and 10% G6PDd prevalence was conducted (at 100% access) where the values for each parameter were independently varied between the low and high values noted in Table 1.

Probabilistic sensitivity analysis (PSA) was conducted using Monte Carlo analysis with 10,000 trials for each scenario. From these results the median number of severe haemolysis events and *P*. *vivax* recurrences, and corresponding trimmed range (10^th^– 90^th^ percentiles), were calculated. Outcomes are expressed per 10,000 *P*. *vivax* patients treated.

### Adherence to primaquine treatment

Current evidence informing the model for the number of recurrences following low- and intermediate-dose PQ treatment is based on efficacy studies where 26%, 69% and 5% had fully supervised, partially supervised and unsupervised treatment, respectively [18]. In routine care it is likely that adherence to the full course of prescribed PQ treatment is less than that in a trial setting, and that adherence decreases with increasing length of treatment. Poor adherence has been shown to increase the risk of recurrence in the first 90 days of treatment [24].

Additional simulations of the model were conducted to explore the impact of reduced adherence, thus better reflecting what may occur in routine care. In these additional simulations it was assumed that all patients prescribed daily PQ take at least the first 3 doses, after which adherence reduces. Severe haemolysis events are related to the daily PQ dose and typically occur within 2–3 days of commencement of treatment [25]. Therefore, poor adherence is assumed not to impact the incidence of severe haemolysis. However, it does impact the expected number of *P*. *vivax* recurrences following treatment due to a reduction in the total amount of PQ consumed [24]. Since there is currently no robust evidence on the relationship between number of *P*. *vivax* recurrences following treatment with total PQ dose <2 mg/kg, a linear relationship was assumed using data for the number of recurrences following no PQ treatment (adherence = 0.0) and low-dose and intermediate-dose PQ (adherence = 1.0). The number of recurrences per person in the 6 months following daily PQ was calculated as ~*Norm*(1.840 − 1.370 × *adherence*, 0.055 − 0.030 × *adherence*)/2.

A similar approach was used for the 8-wk PQ regiment where perfect adherence results in a mean 0.78 recurrences per person-yr and no adherence results in a mean of 1.84 recurrences per person-yr. The number of recurrences per person in the 6 months following weekly PQ was calculated as ~*Norm*(1.840–1.055 × *adherence*, 0.055 − 0.023 × *adherence*)/2.

When investigating the impact of adherence it was assumed that adherence for the intermediate-dose PQ (7 days) ≥ low-dose PQ (14 days) > 8-wk PQ. For simplicity, adherence to the 8-wk PQ regiment was assumed to be 0.2 during the additional simulations, while adherence to low-dose and intermediate-dose PQ were varied between 0.6 and 1.0. Reduced adherence was applied to the use of a specific PQ regiment, irrespective of whether that regiment formed part of the change scenario or baseline. For example, when considering adherence to low-dose PQ any reduction in adherence was applied to both the G6PD-guided low-dose PQ strategy and the baseline strategy (low-dose PQ without G6PD testing).

## Results

### Baseline scenario (Perfect adherence to low-dose PQ for all *P*. *vivax* patients without G6PD testing)

The median number of severe haemolysis events ranged from 15 per 10,000 male *P*. *vivax* patients at 1% G6PDd to 96 per 10,000 male *P*. *vivax* patients at 10% G6PDd for the baseline scenario. This was coupled with a median of 2,363 and 2,418 *P*. *vivax* recurrences within 6 months of treatment at 1% and 10% G6PDd, respectively. Female patients had less than half the number of severe haemolysis events compared to males (median 7 vs 15 at 1% G6PDd; 29 vs 96 at 10% G6PDd), but similar numbers of recurrences (median 2,357 vs 2,363 at 1% G6PDd; median 2,372 vs 2,418 at 10% G6PDd).

### Changes in outcomes when 100% of patients have access to qualitative G6PD testing to guide low-dose PQ, compared to baseline, with perfect adherence

**Fig 3:**
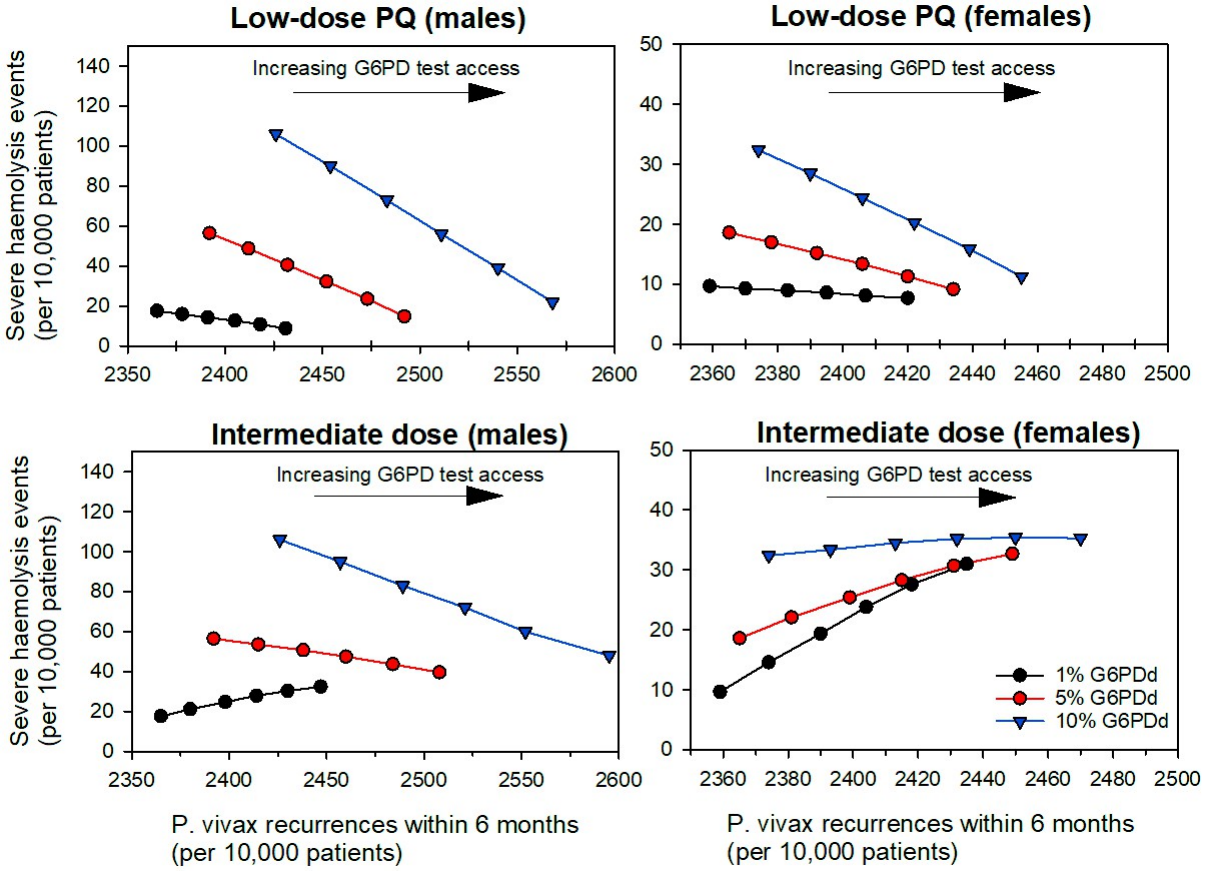
Median number of severe haemolysis events and *P*. *vivax* recurrences per 10,000 male (left) and female (right) *P*. *vivax* patients as access to G6PD testing transitions from 0% (baseline) to 100% for low-dose (top panel) or intermediate-dose PQ scenarios (bottom panel), in 20% increments. Note the different axis scaling for males and females.

The use of qualitative G6PD testing to guide low-dose PQ treatment for all patients reduced the median number of severe haemolysis events and increased the median number of *P*. *vivax* recurrences, compared to baseline for both male and female patients (Fig 3). In PSA, 100% of simulations showed a reduction in severe haemolysis events coupled with an increase in recurrences (Tables 2 and S1).

**Table 2:** PSA comparison of low-dose PQ informed by G6PD testing (100% access) to low-dose PQ used without G6PD testing, assuming 100% adherence to the low-dose PQ regiment.

G6PDd prevalence	Sex	Median change in no. SH events per 10,000 patients (10^th^– 90^th^ percentile)	Median change in no. recurrences per 10,000 patients (10^th^– 90^th^ percentile)	Percent of simulations (n = 10,000)			
				More SH; more recurrences	More SH; fewer recurrences	Fewer SH; more recurrences	Fewer SH; fewer recurrences
1%	Male	-6.4 (-20.4 –-1.3)	64.4 (41.9–95.3)	0.0	0.0	100.0	0.0
	Female	-0.7 (-5.5 –-0.1)	58.1 (37.6–86.5)	0.0	0.0	100.0	0.0
5%	Male	-39.3 (-71.2 –-19.1)	96.0 (64.2–139.7)	0.0	0.0	100.0	0.0
	Female	-7.0 (-22.0 –-1.5)	65.4 (42.5–96.6)	0.0	0.0	100.0	0.0
10%	Male	-81.4 (-128.8 –-47.1)	136.8 (92.3–194.9)	0.0	0.0	100.0	0.0
	Female	-18.5 (-40.9 –-6.9)	76.8 (50.3–112.9)	0.0	0.0	100.0	0.0

SH: severe haemolysis

For males, use of low-dose PQ informed by qualitative G6PD testing decreased the predicted number of severe haemolysis events compared to baseline by 42% and 80%, and increased the number of *P*. *vivax* recurrences within 6 months by 3% and 6%, at 1% and 10% G6PDd respectively (Fig 3, S1 Table). For females there was a 6% and 63% reduction in severe haemolysis events at 1% and 10% G6PDd, respectively. This was coupled with a 3% increase in recurrences (S1 Table).

**Fig 4:**
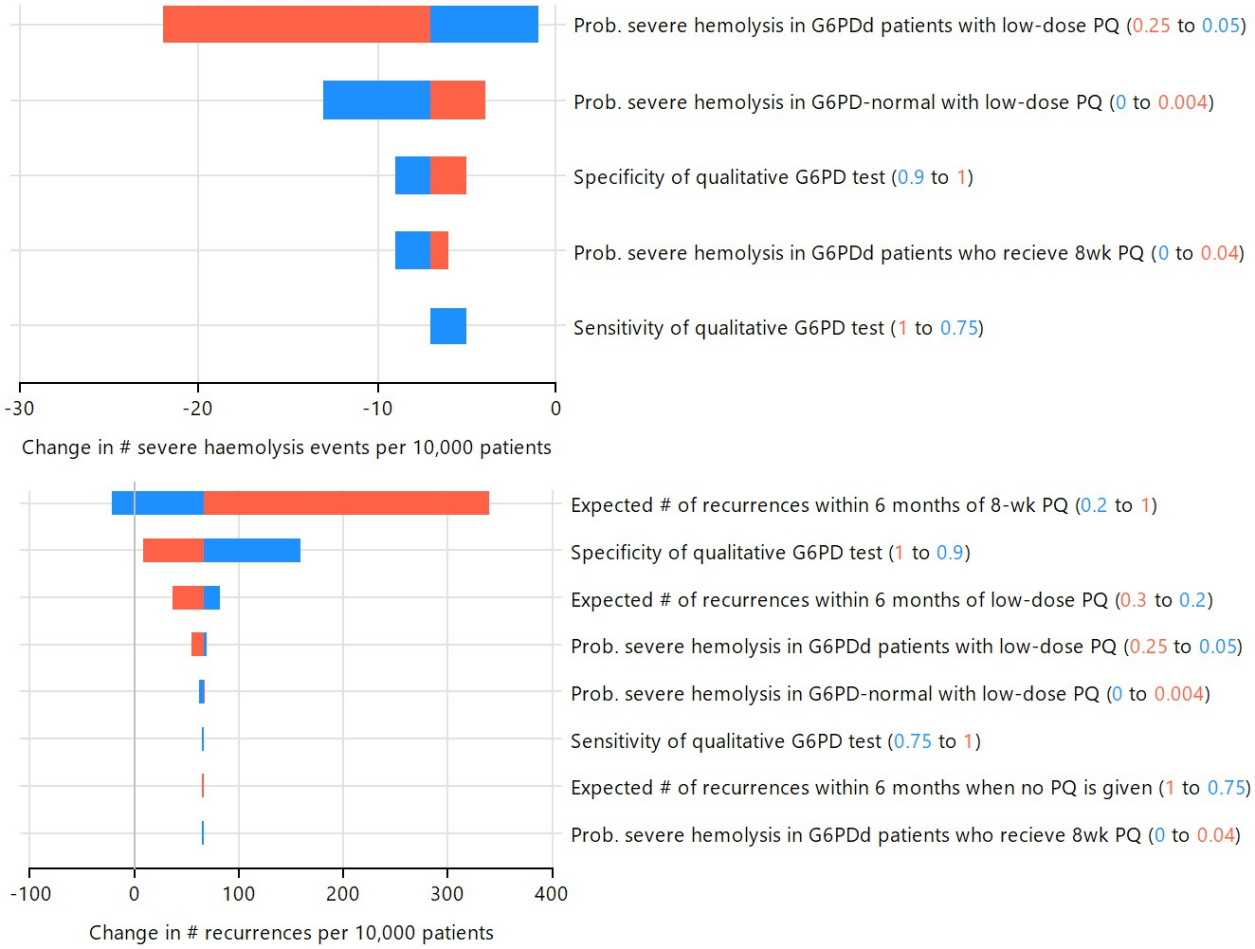
One-way sensitivity analysis of low-dose PQ treatment guided by G6PD testing (100% access), compared to low-dose PQ without G6PD testing. Values for each variable were set at the minimum (blue) and maximum (red) values in independent simulations to investigate the impact on model predictions for number of severe haemolysis events (top) and *P*. *vivax* recurrences within 6 months of treatment (bottom) in male patients with 1% G6PDd prevalence. Only variables impacting each outcome are shown.

The one-way sensitivity analysis indicated the expected number of recurrences associated with 8-wk PQ was the most important variable influencing the change in the number of *P*. *vivax* recurrences within 6 months of treatment when low-dose PQ treatment is guided by G6PD testing compared to low-dose PQ with no G6PD testing; increasing the expected number of recurrences associated with 8-wk PQ led to more *P*. *vivax* recurrences at the population level (Figs 4 and S1). The specificity of the G6PD test also influenced the change in the number of recurrences, with increased specificity leading to fewer recurrences (Figs 4 and S1). In the model, increased specificity reduces the number of G6PD-normal patients who receive 8-wk PQ, leading to fewer *P*. *vivax* recurrences.

At 1% and 10% G6PDd prevalence one-way sensitivity analysis showed the number of severe haemolysis events was most sensitive to the risk of haemolysis for G6PD-defienct patients treated with low-dose PQ (Figs 4 and S1). At 1% G6PDd prevalence the specificity of the G6PD test had a larger impact than the sensitivity on the change in the number of severe haemolysis events, however at 10% G6PD prevalence the G6PD test sensitivity was more influential than specificity (Figs 4 and S1).

### Changes in outcomes when 100% of patients have access to qualitative G6PD testing to guide intermediate-dose PQ, compared to baseline, with perfect adherence to all PQ regiments

**Fig 5:**
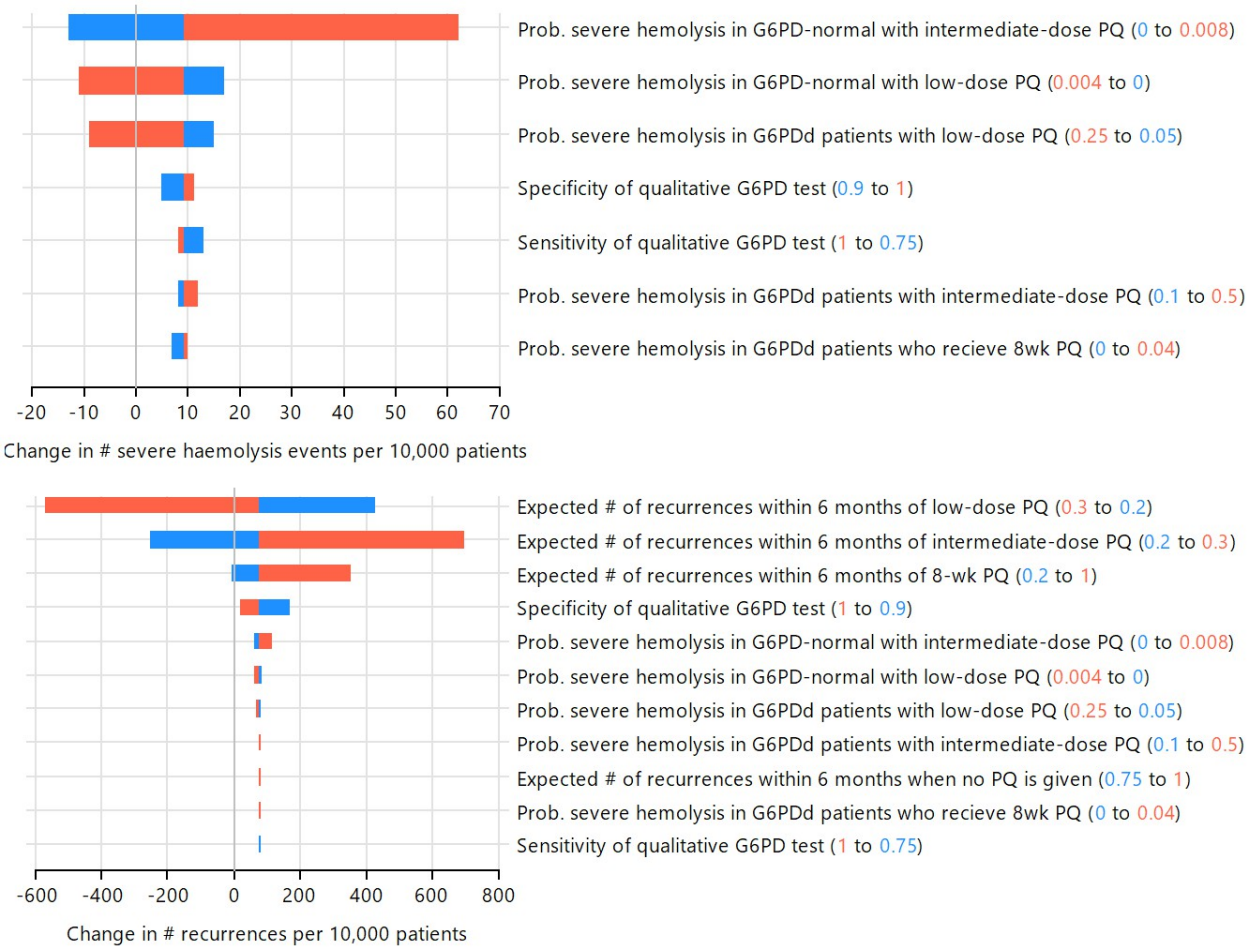
One-way sensitivity analysis of intermediate-dose PQ treatment guided by G6PD testing(100% access), compared to low-dose PQ without G6PD testing. Values for each variable were set at the minimum (blue) and maximum (red) values in independent simulations to investigate the impact on model predictions for number of severe haemolysis events (top) and *P*. *vivax* recurrences within 6 months of treatment (bottom) in male patients with 1% G6PDd prevalence. Only variables impacting each outcome are shown.

Changes in treatment outcomes when qualitative G6PD testing is used to guide intermediate-dose PQ compared to baseline (low-dose PQ for all patients) were mixed, dependent on G6PDd prevalence and sex. In all prevalence scenarios PSA showed the median number of recurrences increased, compared to baseline (Fig 3, S1 Table), but there was wide variability (Table 3). One-way sensitivity analysis indicated that at 1% G6PDd prevalence the expected number of recurrences following low-dose and intermediate-dose PQ were the most important variables influencing the population change in the total number of *P*. *vivax* recurrences within 6 months of treatment (Fig 5). Increasing the expected number of recurrences associated with the intermediate-dose PQ resulted in an increase in the number of recurrences within the patient population for the intermediate-dose PQ strategy compared to baseline. In contrast, increasing the expected number of recurrences associated with low-dose PQ resulted in an overall reduction in the number of recurrences within the population for the intermediate-dose PQ strategy compared to low-dose. At 10% G6PDd, the expected number of recurrences following 8-wk PQ was equally influential (S2 Fig).

**Table 3:** PSA comparison of outcomes for intermediate-dose PQ informed by G6PD testing (100% access) compared to low-dose PQ used without G6PD testing, assuming 100% adherence to the allocated PQ regiment.

G6PDd prevalence	Sex	Median change in no. SH events per 10,000 patients (10^th^– 90^th^ percentiles)	Median change in no. recurrences per 10,000 patients (10^th^– 90^th^ percentiles)	Percent of simulations (n = 10,000)			
				More SH; more recurrences	More SH; fewer recurrences	Fewer SH; more recurrences	Fewer SH; fewer recurrences
1%	Male	13.9 (-10.9–37.4)	82.1 (-140.6–305.0)	53.7	24.1	14.7	7.6
	Female	19.9 (-1.7–43.1)	75.5 (-148.1–299.4)	59.3	29.0	7.5	4.2
5%	Male	-17.1 (-54.0–13.4)	116.5 (-105.0–335.5)	18.5	6.2	56.9	18.5
	Female	12.9 (-11.6–37.1)	82.6 (-140.0–305.2)	53.0	23.7	15.7	7.7
10%	Male	-56.5 (-108.3 –-16.2)	158.1 (-60.3–374.5)	2.8	0.5	80.0	16.7
	Female	2.3 (-26.6–28.5)	95.0 (-127.8–316.7)	38.8	15.8	32.5	13.0

SH: severe haemolysis

Increases in the median number of severe haemolysis events when all patients had access to G6PD testing and adhered to the prescribed PQ regiment were recorded for males at 1% G6PDd, and females at 1% and 5% G6PDd, with minimal difference for females at 10% G6PDd (Fig 3, Table 3). The change point from more to fewer severe haemolysis events in the intermediate-dose PQ scenario compared to baseline occurred when approximately 2.5% of the patient population had G6PD activity < 30% of the AMM.

The one-way sensitivity analysis showed the risk of severe haemolysis in G6PD-normal patients treated with intermediate-dose PQ (positive association), followed by the risk of severe haemolysis in G6PD-deficient and G6PD-normal patients with low-dose PQ (inverse association) were the most influential variables at 1% G6PDd (Fig 5). When the G6PDd prevalence increased to 10% the population change in the number of severe haemolysis events was most influenced by the risk of severe haemolysis in G6PD-deficient patients treated with low-dose PQ (S2 Fig). This highlights the sensitivity of the population-level outcomes to the underlying prevalence of G6PDd amongst *P*. *vivax* patients.

### Effect of reduced adherence to primaquine treatment

The results presented above assume 100% adherence to all PQ regiments. Additional simulations were conducted to investigate the potential effect of lower adherence rates. The assumed adherence rate for a PQ regiment was applied to both the G6PD testing scenario and baseline scenario to ensure a just comparison. Since adherence is assumed to impact the number of recurrences but not severe haemolysis events, results for severe haemolysis are unchanged from those presented above and for brevity have not been repeated.

**Fig 6:**
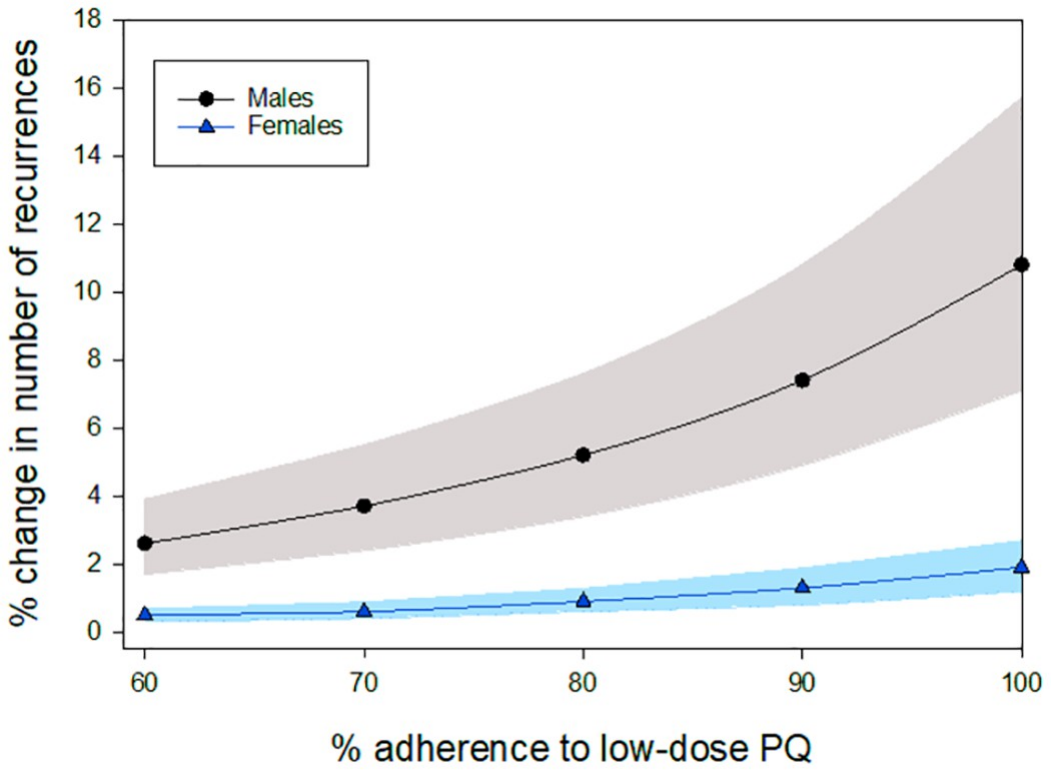
Percentage change in the number of recurrences within 6 months of treatment for low-dose PQ guided by G6PD testing in 100% of patients versus baseline (low-dose PQ without G6PD testing) in a population with 1% G6PDd among *P*. *vivax* patients. Shaded regions represent 10^th^– 90^th^ percentile from PCA.

Reduced adherence to low-dose PQ increased the overall number of *P*. *vivax* recurrences within 6 months of treatment for both the G6PD testing and baseline scenarios, with the largest impact being felt in the baseline scenario where all patients received low-dose PQ. In the G6PD testing scenario a proportion of patients receive 8-wk PQ, so the recurrences in this sub-population were not impacted by changes in adherence to low-dose PQ. As a result, there was a reduction in magnitude of the predicted increase in the total number of *P*. *vivax* recurrences within 6 months of treatment that occurred when 100% of patients had access to G6PD testing, compared to no G6PD testing (Figs 6, S3 and S3 Table).

**Fig 7:**
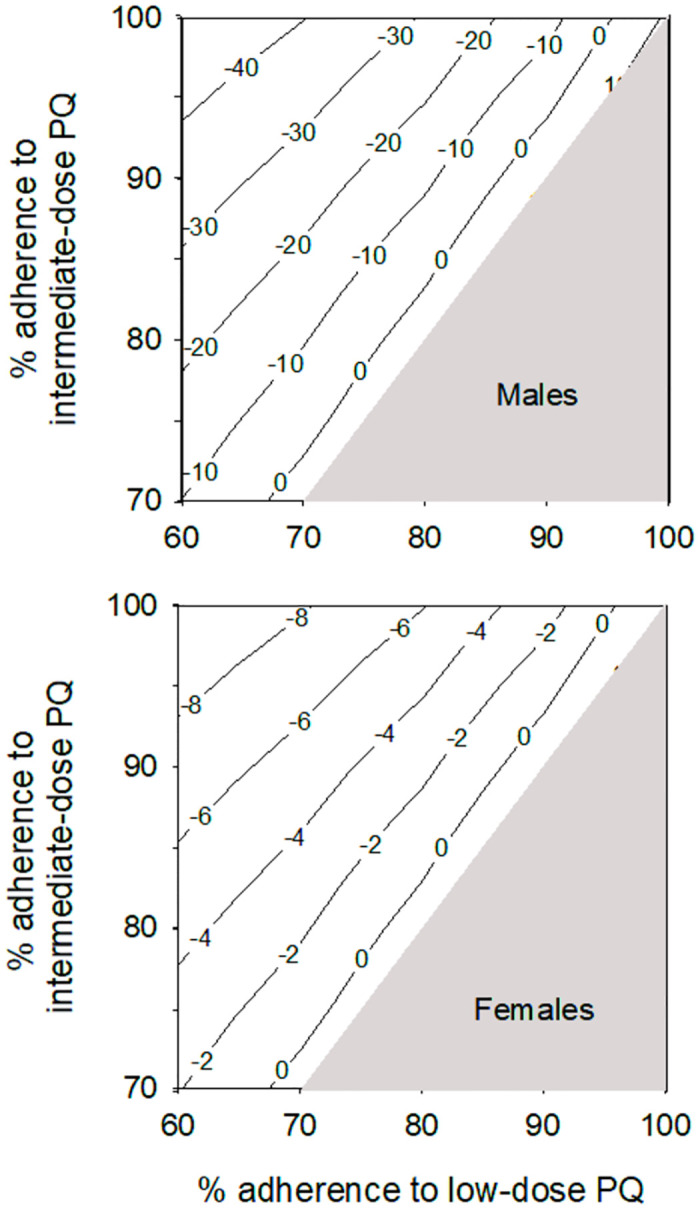
Contour map showing percentage change in median number of *P*. *vivax* recurrences within 6 months of treatment for intermediate-dose PQ guided by G6PD testing (100% access) versus baseline (low-dose PQ without G6PD testing) for varying levels of adherence to low-dose and intermediate-dose PQ regiments in a patient population with 1% G6PDd. It is assumed that adherence to the intermediate-dose PQ regiment is always the same or better than adherence to low-dose PQ.

For the comparison between the intermediate-dose PQ strategy where 100% of patients receive a G6PD test and baseline (low-dose PQ without G6PD testing), reduced adherence increased the number of *P*. *vivax* recurrences within 6 months of treatment for both scenarios. At 1% G6PDd the number of recurrences was predicted to be less in the G6PD testing scenario than baseline when the adherence to the intermediate-dose PQ regiment was at least 5% higher than for the low-dose PQ regiment (Fig 7). A slightly larger difference of approximately 10% in adherence was required for the intermediate-dose PQ strategy to produce fewer *P*. *vivax* recurrences than the baseline scenario at higher G6PDd prevalence (S4 Fig).

## Discussion

This linked-evidence model was created to investigate the downstream impacts of G6PD testing to guide PQ treatment, compared to prescribing PQ without consideration of the G6PD status of the patient. It is based on available evidence, primarily from systematic reviews or meta-analyses. For this reason, the results are generic and not representative of a specific location. Rather, the focus is to investigate generalised patterns and trends, in comparison to a baseline scenario using a hypothesised population of 10,000 *P*. *vivax* patients.

Where low-dose PQ is used without regard to the G6PD status of the patient and adherence to the PQ regiment is perfect (the baseline scenario), introduction of G6PD testing reduces the number of severe haemolysis events in both male and female *P*. *vivax* patients, with a larger impact on the male population due to the higher proportion of patients with G6PD activity <30%. A reduction in severe haemolysis events is expected as most G6PD-deficient patients are administered 8-wk PQ rather than low-dose PQ as a result of applying the G6PD test. This reduction in severe haemolysis is coupled by an increase in the number of *P*. *vivax* recurrences experienced within 6 months of treatment. These changes occur irrespective of G6PDd prevalence. The predicted increase in recurrences can be explained by the sub-population of patients returning positive G6PD tests being treated with 8-wk PQ, which has an assumed lower protective effect than 14-day PQ. The additional recurrences within this sub-population are not completely offset by the recurrences prevented when G6PD-deficient patients experience severe haemolysis and cease treatment in the baseline scenario.

Both G6PD test sensitivity and specificity influence the number of severe haemolysis events, with specificity being more influential at low prevalence of G6PDd. An explanation for this result rests with the model assumption that G6PD-normal patients who receive 8-wk PQ do not experience severe haemolysis, whereas there is a small risk of severe haemolysis with the low-dose PQ regiment. Lower G6PD test specificity increases the number of G6PD-normal patients receiving 8-wk PQ, thus removing them from potentially experiencing severe haemolysis. In contrast, G6PD test sensitivity defines the proportion of G6PD-deficient patients receiving 8-wk PQ, with higher sensitivity resulting in fewer G6PD-deficient patients receiving low-dose PQ. The relative importance of G6PD test sensitivity and specificity to the overall number of severe haemolysis events saved is determined by the ratio of G6PD-deficient to G6PD-normal patients, which is governed by G6PDd prevalence.

In the model, use of intermediate-dose PQ guided by G6PD testing showed variable results, dependent on the proportion of the patient population with G6PD activity <30% of the AMM. With perfect adherence to all PQ regiments reductions in the number of severe haemolysis events from the baseline scenario only occurred when more than 2.5% of the patient population had G6PD activity <30% of the AMM. This pattern can be explained by the competing factors influencing the overall number of severe haemolysis events within the simulated population. The increase in severe haemolysis events at low G6PDd prevalence can be explained by most patients being prescribed intermediate-dose PQ compared to low-dose PQ and the slightly higher risk of severe haemolysis with intermediate-dose PQ in G6PD-normal patients (Table 1). Although the individual risk of severe haemolysis with intermediate-dose PQ is only marginally higher than low-dose PQ, the large number of G6PD-normal individuals receiving this treatment creates more additional severe haemolysis events compared to the severe haemolysis events saved by prescribing G6PD-deficient patients the 8-wk PQ regiment over the low-dose PQ regiment (baseline). As the G6PDd prevalence increases, the reduction in severe haemolysis in this sub-population outweighs the additional severe haemolysis events among G6PD-normal patients, resulting in an overall reduction in severe haemolysis events.

The WHO recommendation for the use of 0.5 mg/kg PQ for 7 days is based on the premise that the shorter duration of treatment will have better adherence, compared to the standard 14-day PQ treatment [4]. The results from this modelling demonstrate that use of the intermediate-dose PQ regiment for patients who test G6PD-normal, coupled with 8-wk PQ for those identified as G6PD-deficient, is predicted to produce fewer recurrences than the low-dose PQ regiment for all patients when the adherence to 7-day PQ is 5–10% higher than adherence to 14-day PQ.

Although radical cure of *P*. *vivax* has been implemented in some countries with or without G6PD testing, many health workers and policymakers are reluctant to prescribe PQ due to concerns about the side-effects, especially when the G6PD status of the patient cannot be readily ascertained [26]. The current study does not consider the scenario where no PQ is used as there is existing evidence supporting the positive impact of adding PQ into the clinical pathway for treatment of *P*. *vivax* [25,27,28]. Instead, this study focuses on the downstream impacts of adding near-patient qualitative G6PD testing into the clinical pathway that already uses PQ and demonstrates that currently available near-patient G6PD tests have sufficient performance to positively impact patient outcomes, particularly in settings with higher prevalence of G6PDd.

The predictions from the model hinge on the quality of the evidence used to inform the parameter values. Data related to the expected number of recurrences among patients receiving 7-day and 14-day PQ was sourced from a meta-analysis based on total PQ prescribed [18]. The same estimate was used for both regiments since the total amount of PQ prescribed is the same. The estimate of recurrence was derived from studies where 58% of patients received 14-day PQ and 42% receive 7-day PQ, but the difference in the recurrence rate between the treatment durations was not tested [18]. It is also unclear whether the expected number of recurrences within 6 months of PQ treatment incorporates repeat PQ treatment for a recurrence, or whether PQ was only used for the initial treatment.

Data on the risk of severe haemolysis following 7-day and 14-day PQ among G6PD-normal patients, and following 14-day PQ and 8-wk PQ amongst G6PD-deficient patients were key variables in determining the population-level change in severe haemolysis events created by adding G6PD testing into the clinical pathway. Data for G6PD-normal patients was sourced from a meta-analysis using the same definition of severe haemolysis as used in this study [21]. However, the data relating to G6PD-deficient patients is less robust with no pre-defined or consistent definition of severe haemolysis, and adverse events not differentiated by PQ dose [3]. It is unlikely that future data on risk of severe haemolysis for G6PD-deficient patients taking 7-day or 14-day PQ will become available given the known adverse effects. Literature exists detailing severe adverse events, including severe haemolysis, when G6PD-deficient patients are accidentally treated with PQ in efficacy studies, but the numbers are small and the denominator is typically unknown, making it difficult to provide robust estimates for the risk of severe haemolysis in this cohort [29–32]. There is also a paucity of evidence on the rate of severe haemolysis for the 8-wk PQ regiment, resulting in the assumption that G6PD-normal patients do not experience severe haemolysis with this treatment, while G6PDd-deficient patients have a rate of severe haemolysis 10-fold lower than the 14-day PQ regiment. The sensitivity analysis indicated that model results for males in higher G6PDd prevalence settings would be most impacted by changes to this assumption.

The specificity of the G6PD test impacted both outcomes but had a larger impact on the number of recurrences. Model output for severe haemolysis was sensitive to the G6PD test sensitivity at higher prevalence of G6PDd. There are several points to note regarding the existing data on qualitative G6PD test performance. Field data reporting the specificity of qualitative G6PD tests pool individuals with normal (>70%) and intermediate (30–70%) G6PD activity into a ‘not-deficient’ group. This group is dominated by individuals with normal G6PD activity due to their higher prevalence in the population, and selection bias due to some studies only enrolling males. If it is assumed that G6PD tests have lower specificity in patients with intermediate G6PD activity (due to having G6PD activity closer to the 30% threshold) then the model output for qualitative G6PD testing under-estimates the number of patients who do not receive 7- or 14-day PQ when they should. This would most effect predictions for female patients in higher G6PDd prevalence settings; 16.9% of females are likely to have intermediate G6PD activity when the G6PDd allele frequency is 10% [7].

Another pertinent point about G6PD testing is that studies have found that repeat testing at different times can produce variable results in malaria patients [32,33]. It has been proposed that G6PD production is dynamic, with increased activity during malaria infection [33]. This creates a diagnostic problem where G6PD-deficient individuals may test normal when infected with *P*. *vivax* and be incorrectly prescribed PQ. Such an effect would not be captured in field trials of G6PD test performance as the recruited individuals are typically healthy. Further field trials involving *P*. *vivax*-infected individuals are required to ensure the G6PD test sensitivities in the literature apply to *P*. *vivax* patients.

Finally, the model only considers severe haemolysis events, not milder haemolysis. Thus, the results should not be considered indicative of all adverse effects from PQ treatment. Likewise, the severity of subsequent *P*. *vivax* recurrences was not considered due to a lack of data. However, it seems logical that a proportion of future *P*. *vivax* recurrences may result in severe disease and that any reduction in the total number of recurrences would ultimately result in a reduction in future severe disease.

Although this study focuses on qualitative G6PD tests, the results would be applicable for near-patient semi-quantitative G6PD tests (at 30% threshold) if they have similar sensitivity and specificity to that used in the model. However, the more refined treatment protocols that are possible using semi-quantitative G6PD tests warrant further investigation, as does G6PD testing with a 70% threshold to guide tafenoquine use.

This study used a decision tree and available evidence to predict two selected health impacts of using qualitative G6PD tests to guide low- or intermediate-dose PQ treatment for radical cure of *P*. *vivax*, compared to using low-dose PQ without G6PD testing. The results generally illustrate reduced severe haemolysis events coupled with small increases in the number of *P*. *vivax* recurrences, although this was not applicable to all G6PDd prevalence rates for the intermediate-dose PQ regiment. The results also highlight the importance of supportive approaches to increase PQ adherence.

## Supporting information

S1 Table Percentage change in severe haemolysis and *P*. *vivax* recurrences in PSA comparison of low-dose and intermediate-dose PQ informed by G6PD testing (100% access) versus low-dose PQ used without G6PD testing, assuming 100% adherence to the assigned treatment PQ regiment. (DOCX)

S2 Table Model outcomes for qualitative G6PD testing to guide low-dose PQ treatment for male and female *P*. *vivax* patients for different levels of access to G6PD testing. Patients who do not receive a G6PD test are treated with low-dose PQ.
(DOCX)

S3 Table Model outcomes for qualitative G6PD testing to guide intermediate-dose PQ treatment for male and female *P*. *vivax* patients for different levels of access to G6PD testing. Patients who do not receive a G6PD test are treated with low-dose PQ.
(DOCX)

S4 Table PSA comparison of low-dose PQ informed by G6PD testing to low-dose PQ used without G6PD testing, for varying levels of PQ adherence and 1% G6PDd. Adherence to the 8-wk PQ regiment is assumed to be 20.
(DOCX)

S1 Fig One-way sensitivity analysis investigating impact of variables on change in number of severe haemolysis events (top) and *P*. *vivax* recurrences within 6 months of treatment (bottom) for low-dose PQ treatment guided by G6PD testing, compared to low-dose PQ without G6PD testing, in male patients when G6PDd prevalence is 10%. Only variables impacting each outcome are shown.
(TIF)

S2 Fig One-way sensitivity analysis investigating impact of variables on change in number of severe haemolysis events (top) and *P*. *vivax* recurrences within 6 months of treatment (bottom) for intermediate-dose PQ treatment guided by G6PD testing, compared to low-dose PQ without G6PD testing, in male patients when G6PDd prevalence is 10%. Only variables impacting each outcome are shown.
(TIF)

S3 Fig Percentage change in the number of recurrences within 6 months of treatment for intermediate-dose PQ versus baseline in a population with 10% G6Pd among *P. vivax* patients. Shaded regions represent 10th–90th percentile from PCA.
(TIF)

S4 Fig Contour map showing percentage change in median number of *P*. *vivax* recurrences within 6 months of treatment for intermediate-dose PQ versus baseline for varying levels of adherence to low-dose and intermediate-dose PQ regiments in a patient population with 10% G6PDd. It is assumed that adherence to intermediate-dose PQ is always the same or better than adherence to low-dose PQ.
(TIF)
