# Supplementary material for: Linked-evidence modelling of qualitative G6PD testing to inform low- and intermediate-dose primaquine treatment for radical cure of Plasmodium vivax
Source: PLoS Negl Trop Dis. 2024 Sep 5;18(9):e0012486. doi: 10.1371/journal.pntd.0012486 (PMC11407642; doi:10.1371/journal.pntd.0012486)
Supplement: S1 Table — (DOCX) [file pntd.0012486.s001.docx]

S1 Table. Percentage change in severe haemolysis and *P. vivax* recurrences in PSA comparison of low-dose and intermediate-dose PQ informed by G6PD testing (100% access) versus low-dose PQ used without G6PD testing, assuming 100% adherence to the assigned treatment PQ regiment

| G6PDd prevalence | Sex | Low-dose PQ | | Intermediate-dose PQ | |
| --- | --- | --- | --- | --- | --- |
|  |  | Median percentage change^a^ in no. SH events per 10,000 patients (10^th^ – 90^th^ percentile) | Median percentage change^a^ in no. recurrences per 10,000 patients (10^th^ – 90^th^ percentile) | Median percentage change^a^ in no. SH events per 10,000 patients (10^th^ – 90^th^ percentile) | Median percentage change^a^ in no. recurrences per 10,000 patients (10^th^ – 90^th^ percentile) |
| 1% | Male | -42.2 (-77.3 – -11.0) | 2.7 (1.7 – 4.1) | 84.5 (-31.1 – 528.4) | 3.5 (-5.7 – 13.5) |
|  | Female | -6.3 (-49.0 – -3.1) | 2.5 (1.6 – 3.8) | 224.4 (-7.7 – 1,749.8) | 3.2 (-6.0 – 13.3) |
| 5% | Male | -73.2 (-87.4 – -49.5) | 4.0 (2.6 – 6.0) | -30.8 (-63.3 – 37.3) | 4.9 (-4.2 – 14.7) |
|  | Female | -44.9 (-78.1 – -12.4) | 2.8 (1.8 – 4.2) | 72.8 (-33.3 – 466.8) | 3.5 (-5.6 – 13.6) |
| 10% | Male | -79.5 (-89.6 – -61.5) | 5.6 (3.7 – 8.2) | -54.4 (-73.6 – -21.9) | 6.5 (-2.4 – 16.2) |
|  | Female | -63.2 (-84.1 – -31.9) | 3.2 (2.1 – 4.9) | 7.5 (-49.7 – 160.5) | 4.0 (-5.1 – 14.0) |

^a^ Percentage change calculated as G6PD-informed scenario / baseline scenario * 100. Percentage change calculated for each simulation in PCA, then median and percentiles were determined.
