# Supplementary material for: Linked-evidence modelling of qualitative G6PD testing to inform low- and intermediate-dose primaquine treatment for radical cure of Plasmodium vivax
Source: PLoS Negl Trop Dis. 2024 Sep 5;18(9):e0012486. doi: 10.1371/journal.pntd.0012486 (PMC11407642; doi:10.1371/journal.pntd.0012486)
Supplement: S2 Table — Patients who do not receive a G6PD test are treated with low-dose PQ. (DOCX) [file pntd.0012486.s002.docx]

S2 Table. Model outcomes for qualitative G6PD testing to guide low-dose PQ treatment for male and female *P. vivax* patients for different levels of access to G6PD testing. Patients who do not receive a G6PD test are treated with low-dose PQ.

| Proportion of patients with access to G6PD test | Qualitative G6PD testing (10,000 male patients) | | Qualitative G6PD testing (10,000 female patients) | |
| --- | --- | --- | --- | --- |
|  | Median severe haemolysis events (trimmed range) | Median recurrences (trimmed range) | Median severe haemolysis events (trimmed range) | Median recurrences (trimmed range) |
| **1% G6PDd** | | | | |
| 0.0 | 17.6 (5.6 – 41.3) | 2364.6 (2203.6 – 2524.3) | 9.7 (1.8 – 28.7) | 2358.8 (2200.0 – 2518.5) |
| 0.2 | 15.9 (5.1 – 37.9) | 2377.9 (2218.5 – 2536.8) | 9.3 (1.8 – 27.8) | 2370.5 (2212.8 – 2529.6) |
| 0.4 | 14.3 (4.5 – 34.7) | 2390.8 (2233.6 – 2549.4) | 9.0 (1.7 – 27.1) | 2382.6 (2225.3 – 2540.8) |
| 0.6 | 12.6 (3.9 – 31.5) | 2404.6 (2247.6 – 2562.1) | 8.6 (1.6 – 26.3) | 2394.6 (2237.5 – 2552.3) |
| 0.8 | 10.8 (3.2 – 28.7) | 2418.1 (2262.1 – 2575.2) | 8.1 (1.5 – 25.6) | 2407.3 (2250.9 – 2563.7) |
| 1.0 | 8.8 (2.2 – 26.2) | 2431.4 (2275.8 – 2588.6) | 7.7 (1.3 – 24.9) | 2419.5 (2263.6 – 2575.9) |
| **5% G6PDd** | | | | |
| 0.0 | 56.5 (30.8 – 94.6) | 2391.9 (2229.9 – 2553.2) | 18.6 (6.2 – 42.3) | 2365.3 (2205.2 – 2525.0) |
| 0.2 | 48.7 (26.4 – 81.4) | 2411.6 (2251.8 – 2570.9) | 17.0 (5.6 – 38.4) | 2378.1 (2221.0 – 2537.1) |
| 0.4 | 40.5 (21.9 – 68.2) | 2432.1 (2271.4 – 2589.3) | 15.2 (4.9 – 34.9) | 2391.8 (2234.8 – 2549.2) |
| 0.6 | 32.2 (17.4 – 55.6) | 2452.3 (2292.8 – 2607.9) | 13.4 (4.2 – 31.5) | 2405.8 (2249.0 – 2562.9) |
| 0.8 | 23.6 (12.2 – 43.9) | 2472.7 (2313.4 – 2627.7) | 11.3 (3.4 – 28.7) | 2419.9 (2263.7 – 2575.8) |
| 1.0 | 14.8 (5.8 – 33.4) | 2492.4 (2333.8 – 2645.9) | 9.2 (2.3- 26.2) | 2433.9 (2277.0 – 2588.3) |
| **10% G6PDd** | | | | |
| 0.0 | 105.6 (66.8 – 159.4) | 2425.7 (2262.2 – 2588.7) | 32.4 (14.4 – 62.0) | 2374.1 (2215.0 – 2534.9) |
| 0.2 | 89.5 (56.8 – 134.4) | 2454.1 (2293.1 – 2614.0) | 28.5 (12.6 – 54.5) | 2390.2 (2231.9 – 2549.8) |
| 0.4 | 73.2 (46.4 – 109.8) | 2482.7 (2323.4 – 2641.0) | 24.4 (10.7 – 47.4) | 2406.2 (2250.1 – 2564.5) |
| 0.6 | 56.3 (35.5 – 85.9) | 2511.4 (2352.7 – 2667.0) | 20.3 (8.7 – 40.5) | 2422.3 (2266.8 – 2580.0) |
| 0.8 | 39.2 (23.7 – 63.7) | 2539.5 (2381.5 – 2693.7) | 15.9 (6.4 – 34.1) | 2438.8 (2282.9 – 2594.9) |
| 1.0 | 21.5 (9.8 – 43.9) | 2567.7 (2409.2 – 2721.6) | 11.3 (3.7 – 28.6) | 2455.0 (2299.4 – 2610.0) |
