# Supplementary material for: Linked-evidence modelling of qualitative G6PD testing to inform low- and intermediate-dose primaquine treatment for radical cure of Plasmodium vivax
Source: PLoS Negl Trop Dis. 2024 Sep 5;18(9):e0012486. doi: 10.1371/journal.pntd.0012486 (PMC11407642; doi:10.1371/journal.pntd.0012486)
Supplement: S3 Table — Patients who do not receive a G6PD test are treated with low-dose PQ. (DOCX) [file pntd.0012486.s003.docx]

S3 Table. Model outcomes for qualitative G6PD testing to guide intermediate-dose PQ treatment for male and female *P. vivax* patients for different levels of access to G6PD testing. Patients who do not receive a G6PD test are treated with low-dose PQ.

| Proportion of patients with access to G6PD test | Qualitative G6PD testing (10,000 male patients) | | Qualitative G6PD testing (10,000 female patients) | |
| --- | --- | --- | --- | --- |
|  | Median severe haemolysis events (trimmed range) | Median recurrences (trimmed range) | Median severe haemolysis events (trimmed range) | Median recurrences (trimmed range) |
| **1% G6PDd** | | | | |
| 0.0 | 17.6 (5.6 – 41.3) | 2364.6 (2203.6 – 2524.3) | 9.7 (1.8 – 28.7) | 2358.8 (2200.0 – 2518.5) |
| 0.2 | 21.1 (10.4 – 40.8) | 2380.4 (2249.8 – 2512.2) | 14.6 (7.1 – 30.4) | 2374.4 (2243.4 – 2504.0) |
| 0.4 | 24.8 (13.8 – 41.6) | 2398.1 (2281.6 – 2510.5) | 19.4 (10.7 – 33.7) | 2389.7 (2274.3 – 2502.1) |
| 0.6 | 27.9 (15.9 – 44.1) | 2414.1 (2300.1 – 2526.9) | 23.8 (13.4 – 38.6) | 2404.2 (2290.5 – 2516.6) |
| 0.8 | 30.3 (17.3 – 48.6) | 2429.8 (2302.0 – 2558.7) | 27.6 (15.2 – 45.2) | 2418.0 (2291.3 – 2548.1) |
| 1.0 | 32.5 (17.6 – 54.01) | 2446.6 (2291.8 – 2601.3) | 31.0 (16.4 – 52.6) | 2434.7 (2279.5 – 2589.9) |
| **5% G6PDd** | | | | |
| 0.0 | 56.5 (30.8 – 94.6) | 2391.9 (2229.9 – 2553.2) | 18.6 (6.2 – 42.3) | 2365.3 (2205.2 – 2525.0) |
| 0.2 | 53.6 (31.9 – 85.2) | 2414.8 (2281.5 – 2548.6) | 22.1 (11.0 – 41.6) | 2381.4 (2251.6 – 2512.3) |
| 0.4 | 50.7 (32.1 – 76.5) | 2438.1 (2321.0 – 2554.3) | 25.4 (14.3 – 42.1) | 2399.3 (2283.8 – 2511.5) |
| 0.6 | 47.5 (31.0 – 69.3) | 2460.5 (2344.9 – 2574.8) | 28.3 (16.5 – 44.5) | 2414.5 (2301.8 – 2528.3) |
| 0.8 | 43.7 (28.5 – 64.3) | 2483.8 (2354.7 – 2612.5) | 30.7 (17.6 – 48.8) | 2431.0 (2302.8 – 2559.7) |
| 1.0 | 39.6 (24.0 – 61.8) | 2508.0 (2353.3 – 2661.6) | 32.7 (17.8 – 54.4) | 2448.6 (2293.1 – 2602.8) |
| **10% G6PDd** | | | | |
| 0.0 | 105.6 (66.8 – 159.4) | 2425.7 (2262.2 – 2588.7) | 32.4 (14.4 – 62.0) | 2374.1 (2215.0 – 2534.9) |
| 0.2 | 94.5 (62.4 – 138.8) | 2457.2 (2320.8 – 2594.1) | 33.4 (19.7 – 57.9) | 2393.4 (2262.6 – 2525.5) |
| 0.4 | 83.4 (57.2 – 119.0) | 2489.0 (2369.1 – 2607.8) | 34.5 (20.5 – 55.0) | 2413.3 (2297.4 – 2526.8) |
| 0.6 | 72.3 (50.6 – 101.0) | 2520.9 (2403.5 – 2636.9) | 35.2 (21.7 – 53.7) | 2431.5 (2317.1 – 2544.9) |
| 0.8 | 60.4 (42.1 – 85.6) | 2551.8 (2422.9 – 2682.0) | 35.4 (21.6 – 54.9) | 2450.1 (2321.9 – 2579.4) |
| 1.0 | 48.1 (30.5 – 73.8) | 2584.8 (2428.9 – 2738.1) | 35.3 (20.1 – 57.4) | 2469.9 (2314.7 – 2624.7) |
