# Supplementary material for: Linked-evidence modelling of qualitative G6PD testing to inform low- and intermediate-dose primaquine treatment for radical cure of Plasmodium vivax
Source: PLoS Negl Trop Dis. 2024 Sep 5;18(9):e0012486. doi: 10.1371/journal.pntd.0012486 (PMC11407642; doi:10.1371/journal.pntd.0012486)
Supplement: S4 Table — Adherence to the 8-wk PQ regiment is assumed to be 20. (DOCX) [file pntd.0012486.s004.docx]

S4 Table. PSA comparison of low-dose PQ informed by G6PD testing to low-dose PQ used without G6PD testing, for varying levels of PQ adherence and 1% G6PDd. Adherence to the 8-wk PQ regiment is assumed to be 20%.

| Adherence to low-dose PQ (%) | Change in no. severe haemolysis events per 10,000 patients (10^th^ – 90^th^ percentiles) | Change in no. recurrences per 10,000 patients (10^th^ – 90^th^ percentiles) | Median percentage change in recurrences (10^th^-90^th^ percentiles) |
| --- | --- | --- | --- |
|  |  |  |  |
| **Males** |  |  |  |
| 100 | -6.4 (-20.4 – -1.3) | 255.0 (168.8 – 369.1) | 10.8 (7.1 – 15.7) |
| 90 | -6.4 (-20.4 – -1.3) | 224.4 (148.7 – 325.9) | 7.4 (4.9 – 10.8) |
| 80 | -6.4 (-20.4 – -1.3) | 194.2 (128.1 – 282.5) | 5.2 (3.4 – 7.6) |
| 70 | -6.4 (-20.4 – -1.3) | 163.6 (107.8 – 238.9) | 3.7 (2.4 – 5.5) |
| 60 | -6.4 (-20.4 – -1.3) | 133.3 (87.1 – 196.4) | 2.6 (1.7 – 3.9) |
| **Females** |  |  |  |
| 100 | -0.1 (-1.1 – -0.02) | 43.8 (29.0 – 63.4) | 1.9 (1.2 – 2.7) |
| 90 | -0.1 (-1.1 – -0.02) | 38.6 (25.6 – 56.0) | 1.3 (0.8 – 1.9) |
| 80 | -0.1 (-1.1 – -0.02) | 33.4 (22.1 – 48.5) | 0.9 (0.6 – 1.3) |
| 70 | -0.1 (-1.1 – -0.02) | 28.1 (18.6 – 41.2) | 0.6 (0.4 – 0.9) |
| 60 | -0.1 (-1.1 – -0.02) | 23.0 (15.1 – 33.8) | 0.5 (0.3 – 0.7) |
