# Supplementary figures and images for: Linked-evidence modelling of qualitative G6PD testing to inform low- and intermediate-dose primaquine treatment for radical cure of Plasmodium vivax
Source: PLoS Negl Trop Dis. 2024 Sep 5;18(9):e0012486. doi: 10.1371/journal.pntd.0012486 (PMC11407642; doi:10.1371/journal.pntd.0012486)

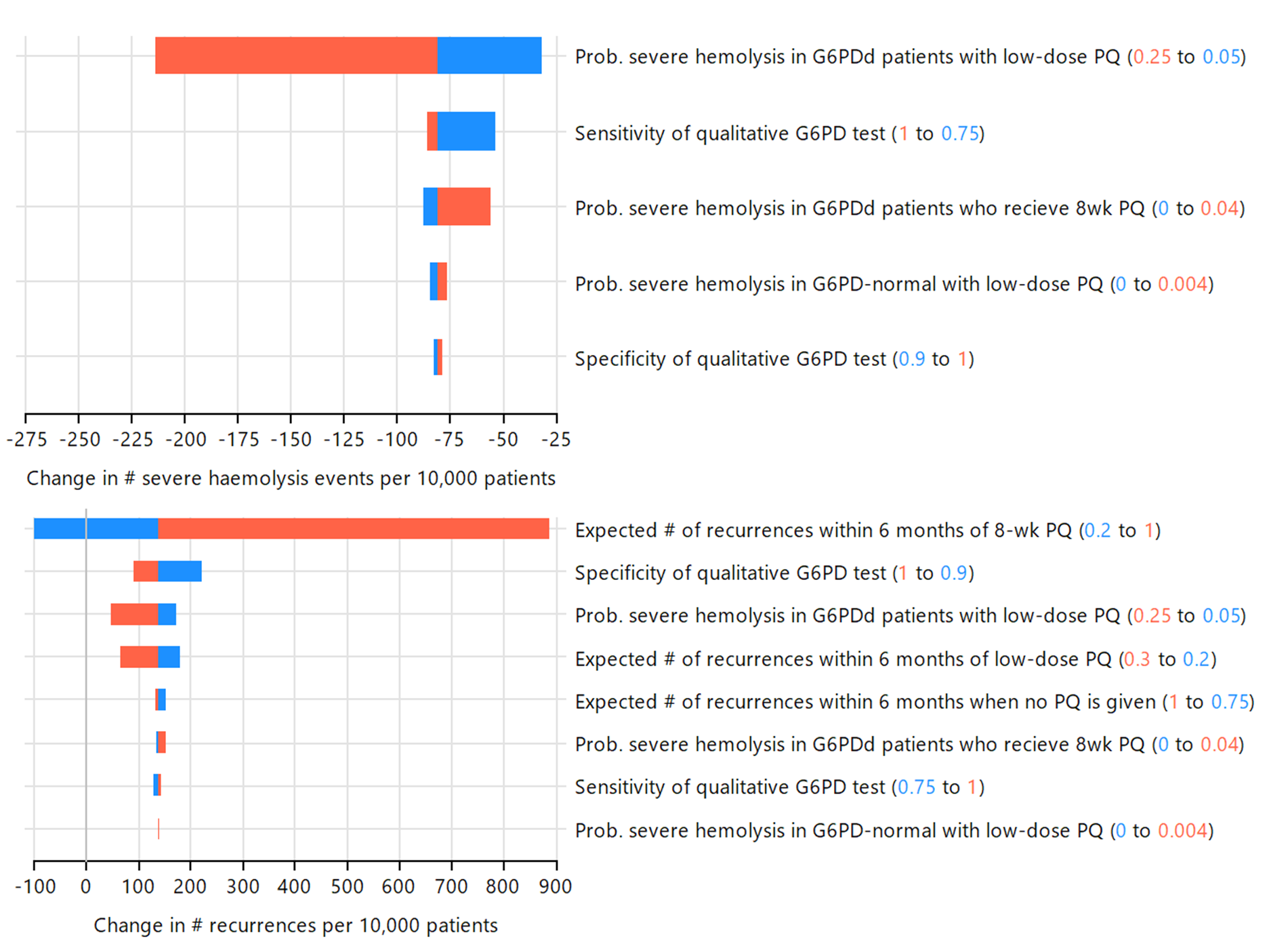

Supplement: S1 Fig — Only variables impacting each outcome are shown. (TIF) [file pntd.0012486.s005.tif]

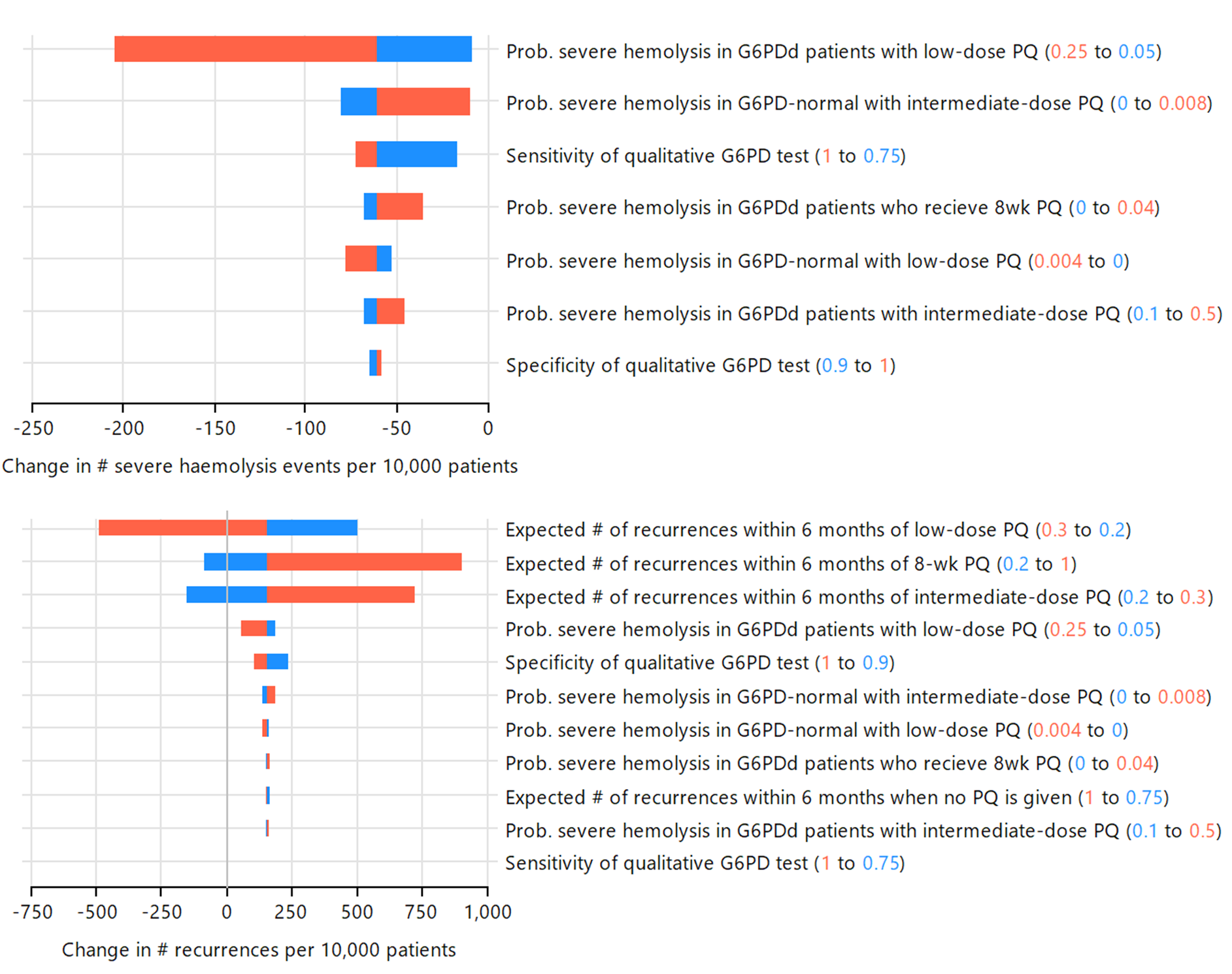

Supplement: S2 Fig — Only variables impacting each outcome are shown. (TIF) [file pntd.0012486.s006.tif]

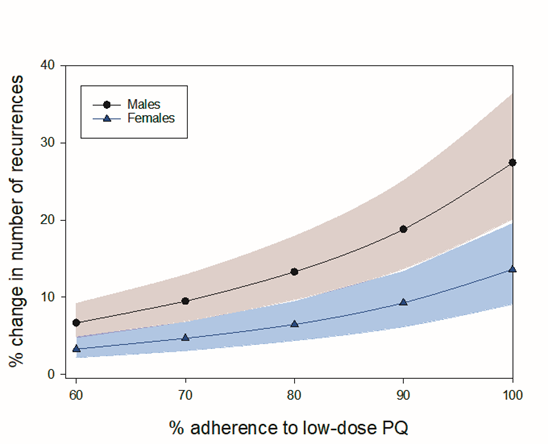

Supplement: S3 Fig — Shaded regions represent 10th–90th percentile from PCA. (TIF) [file pntd.0012486.s007.tif]

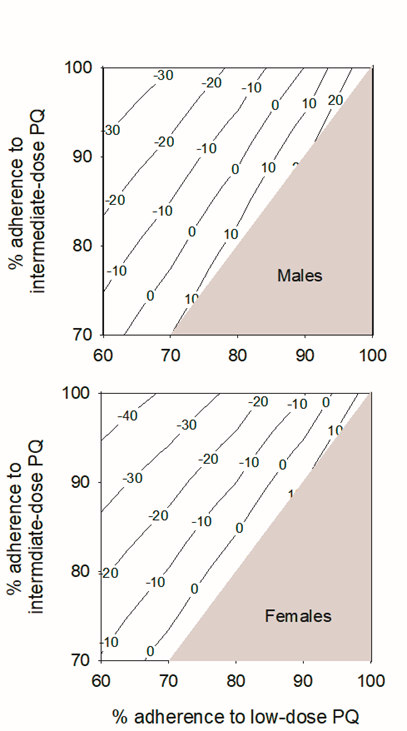

Supplement: S4 Fig — It is assumed that adherence to intermediate-dose PQ is always the same or better than adherence to low-dose PQ. (TIF) [file pntd.0012486.s008.tif]
